# Rational Design of Non-Covalent Imprinted Polymers Based on the Combination of Molecular Dynamics Simulation and Quantum Mechanics Calculations

**DOI:** 10.3390/polym16162257

**Published:** 2024-08-09

**Authors:** Xue Yu, Jiangyang Mo, Mengxia Yan, Jianhui Xin, Xuejun Cao, Jiawen Wu, Junfen Wan

**Affiliations:** 1 Shandong Key Laboratory of Biophysics, Institute of Biophysics, Dezhou University, Dezhou 253023, China; y12180348@mail.ecust.edu.cn (X.Y.); jymo@dzu.edu.cn (J.M.); yanmengxia1219@163.com (M.Y.); jhxin_mxyan@163.com (J.X.); 2 State Key Laboratory of Bioreactor Engineering, Department of Bioengineering, East China University of Science and Technology, 130 Meilong Rd., Shanghai 200237, China; caoxj@ecust.edu.cn; 3 School of Energy and Machinery, Dezhou University, Dezhou 253023, China

**Keywords:** quantum mechanics, rational design, molecular imprinting, molecular dynamics

## Abstract

Molecular imprinting is a promising approach for developing polymeric materials as artificial receptors. However, only a few types of molecularly imprinted polymers (MIPs) are commercially available, and most research on MIP_S_ is still in the experimental phase. The significant limitation has been a challenge for screening imprinting systems, particularly for weak functional target molecules. Herein, a combined method of quantum mechanics (QM) computations and molecular dynamics (MD) simulations was employed to screen an appropriate 2,4-dichlorophenoxyacetic acid (2,4-D) imprinting system. QM calculations were performed using the Gaussian 09 software. MD simulations were conducted using the Gromacs2018.8 software suite. The QM computation results were consistent with those of the MD simulations. In the MD simulations, a realistic model of the ‘actual’ pre-polymerisation mixture was obtained by introducing numerous components in the simulations to thoroughly investigate all non-covalent interactions during imprinting. This study systematically examined MIP systems using computer simulations and established a theoretical prediction model for the affinity and selectivity of MIPs. The combined method of QM computations and MD simulations provides a robust foundation for the rational design of MIPs.

## 1. Introduction

Molecular imprinting technology (MIT) is a polymer-preparation-based synthesis process of artificial receptors for specific target molecules [1,2]. Molecularly imprinted polymers (MIPs) prepared by MIT have the following significant advantages: low price, easy preparation, high selectivity and good reusability [3]. Over the past few decades, MIT has successfully been employed in solid phase extraction [4,5,6], catalysis [7,8], drug delivery systems [9] and sensors [10,11]. However, only a few types of MIPs are commercially available currently, and most research on MIP_S_ is still in the experimental stage due to challenges in screening imprinting systems, particularly for weak functional target molecules.

Common screening methods for imprinting systems include experimental and ultraviolet (UV) spectral methods [12,13]. Traditional experimental approaches primarily rely on numerous experiments and previous research. The accuracy of UV spectral methods is low [14,15]. The introduction of computational chemistry and molecular simulation methods offers a new approach to screening imprinting systems [6,15,16,17]. Quantum mechanics (QM) computations can be employed to determine putative complexes among monomers and templates to obtain the optimal monomer with high selectivity and rebinding capacity to templates [18]. Molecular dynamics (MD) simulations can simulate and predict the properties of an ensemble comprising non-covalent complexes formed between polymer components in a MIP pre-polymerisation mixture [14,19]. There have been a few attempts for combining QM computations with MD simulations to predict the composition of pre-polymerisation mixtures for MIPs [20].

**Figure 1:**
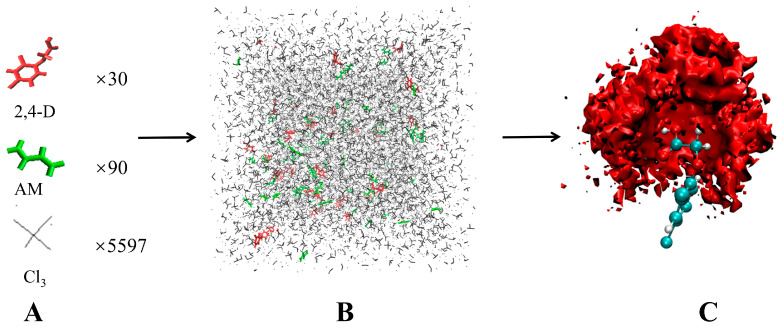
Schematic of the steps involved in a typical molecular dynamics simulation for a pre-polymerisation mixture illustrated for a system based on a functional monomer (AM) and 2,4-D in chloroform: (**A**) Components added to the design. (**B**) A 5 ns production phase and the pre-polymerisation mixture after equilibration. (**C**) Statistical analysis results of the different pre-polymerisation mixtures produced.

Herein, a combined method of QM computations and MD simulations was used to screen appropriate 2,4-dichlorophenoxyacetic acid (2,4-D) imprinting systems. This integrated screening method of imprinting systems leveraged the advantages of the two approaches to provide a more comprehensive description of the imprinting systems. QM computations were performed using the Gaussian 09 software, and MD simulations were performed using the Gromacs2018.8 programme. Among various dynamics programmes, Gromacs provided the highest computational speed and performance and has improved consistently based on recent developments in Graphics Processing Unit (GPU) and Central Processing Unit (CPU) hardware [21,22]. The steps involved in the MD simulations are illustrated in Figure 1. Based on the molecular structure of 2,4-D, 11 imprinting systems were examined, including two imprinting solvents (dimethyl sulfoxide (DMSO) and chloroform) and five functional monomers (acrylamide (AM), 2-(dimethylamino)ethyl methacrylate (DMAEMA), methacrylic acid (MAA), 2-trifluoromethacrylic acid (TFMAA) and 4-vinylpyridine (4-VP)).

## 2. Computational Details

### 2.1. QM Calculations for Characterising Template–Monomer Complexes

All geometric structures were computed using Gaussian 09 software [23] with the polarisable continuum model. The Becke 3-parameter-Lee-Yang-Parr (B3LYP) functional with a 6-311+G** basis set was used for optimisation based on Grimme’s D3 dispersion correction [24,25]. Frequency computations were conducted at the same level to determine whether a stationary point was a minimum at T = 298.15 K. Interaction energy, ΔE, is computed using the following equation:

ΔE = E(template–monomer complex) − E(template) − E(monomer) (1)

where E(template–monomer complex) represents the energy of the complexes, E(template) denotes the energy of 2,4-D and E(monomer) represents the energy of functional monomers.

### 2.2. MD Simulations for the Characterisation of the Types and Strengths of All Pre-Polymerization Interactions

All MD simulations were conducted using the Gromacs2018.8 suite of programmes [21,22]. The general amber force field [26] was used to establish and parameterise the molecular systems. The RESP20.5 charge method was used to assign the partial atomic charges. Multiwfn [27] used a highly efficient algorithm to evaluate the electrostatic potential employed in the analysis [28,29,30]. The system composition is presented in Table 1. Table 1 shows the number of different molecules added during the MD simulations.

Periodic boundary conditions were used in these simulations, and the simulation box’s size was approximately 10^3^ nm^3^. The order of simulation was as follows: 2,4-D (30 molecules) and a functional monomer (90–150 molecules) were added into a small box and solvated with 5597 molecules of chloroform/DMSO. This simulation condition approximately corresponded to 1 mmol of 2,4-D and 3–5 mmol of the functional monomer in 15 mL of solvent. Subsequently, the steepest descent approach was used to eliminate bad van der Waals contacts. Following these energy optimisation procedures, they were annealed for 2 ns under a constant volume at NVT, and temperature variations with simulation time were recorded (Figure S1). The boxes were then equilibrated at NPT (298.15 K, 1 bar) for 3 ns. MD simulation production was performed for 5 ns at the NPT after equilibration of the systems.

For non-bonding interactions, a cut-off of 1.0 nm was used for all the prepared systems. The particle mesh Ewald simulation method was used to handle the electrostatic interactions in the systems. The cut-off simulation method was used to manage the van der Waals forces in these systems. During 5 ns of equilibration, a Berendsen barostat and velocity rescaling thermostat were used to control the pressure and temperature, respectively. During the 5 ns of production, the temperature and pressure were regulated using the Parrinello–Rahman barostat and velocity rescaling thermostat.

**Figure 2:**
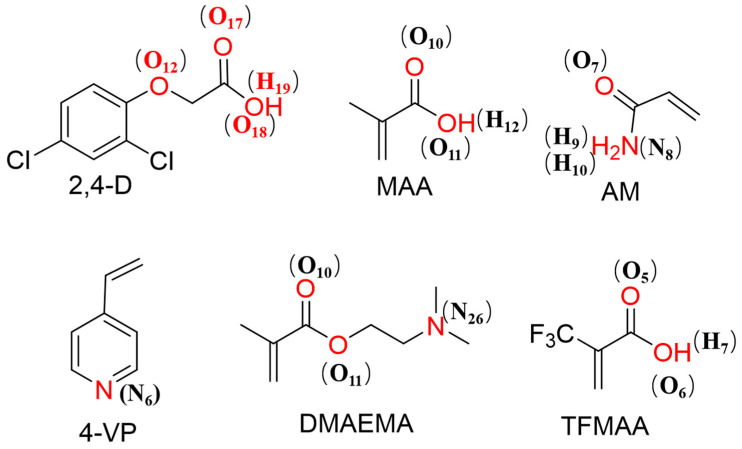
Atoms studied using radial distribution functions (RDFs).

The trajectory and statistical analyses of the simulation data were performed using GROMACS’ practical tools, and the results were visualised using visual molecular dynamics (VMD) [31]. Hydrogen bond, radial distribution functions (RDFs) and spatial distribution functions (SDF) computations were primarily included in the analysis. RDFs were generated to measure the local concentrations of specific atom pairs at optimal interaction distances. The investigated atom pairs were described (Figure 2). Hydrogen bonds in the trajectories were counted using a GROMACS command based on geometric standards. O and N served as H–H bond acceptors and O–H and N–H served as H–H bond donors. The standard of H–H bonds is introduced below. The angle of the H–H donor-acceptor was less than 30° and the distance between the donor and the acceptor was less than 3.5 Å (Figure S2). The SDF was generated using the gmx spatial tool in the GROMACS and VMD packages. First, the trajectory was processed using the gmx spatial tool in GROMACS to create a cube file. To obtain meaningful SDFs, the solute molecules must be centred within the box for the entire trajectory, and the molecules for which SDF computations are required should be grouped and defined. Subsequently, the SDF is visualised using the VMD package.

## 3. Results

### 3.1. QM Calculations for Characterising Template–Monomer Complexes

**Figure 3:**
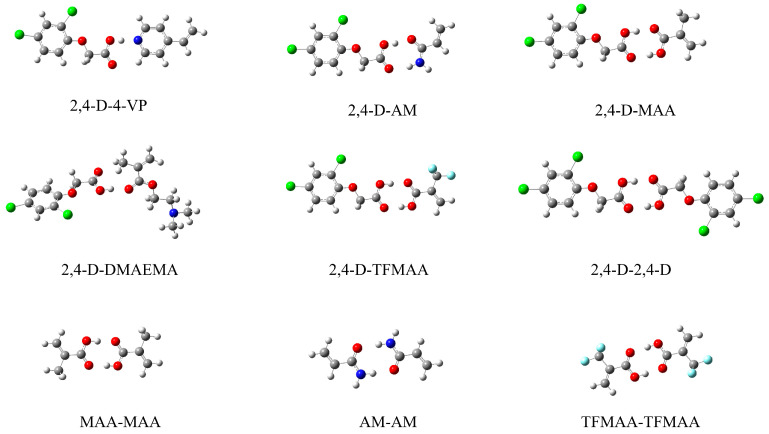
Optimised geometry of complexes; the ab initio mechanical quantum computation was based on Density Function Theory (DFT) at the Becke 3-parameter-Lee-Yang-Parr (B3LYP) level with 6-311+G** basis set.

The optimised conformations of 2,4-D and the five functional monomers were obtained (Figure S3). The optimised conformations of the complexes are illustrated in Figure 3. The interaction energies of the 2,4-D–functional monomer complexes in different solvents are presented in Table 2 and Table 3. As shown in Table 2, the stability of the complexes in chloroform decreased in the following order: MAA (−63.68 kJ/mol) > TFMAA (−63.00 kJ/mol) > AM (−61.82 kJ/mol) > 4-VP (−58.54 kJ/mol) > DMAEMA (−39.31 kJ/mol). As shown in Table 3, the stability order of the complexes in DMSO was as follows: TFMAA (−57.84 kJ/mol) > AM (−56.17 kJ/mol) > 4-VP (−55.56 kJ/mol). When the porogen was changed from CHCl_3_ to the more polar DMSO, the binding stability between 2,4-D and the functional monomers decreased.

The same method was used to determine the binding energies of 2,4-D–2,4-D, MAA–MAA and TFMAA–TFMAA in chloroform (Table 2). The binding energies of TFMAA–TFMAA and MAA–MAA were −64.61 and −64.05 kJ/mol, respectively, which were higher than those of 2,4-D–TFMAA (−63.00 kJ/mol) and 2,4-D–MAA (−63.68 kJ/mol). Thus, using MAA or TFMAA as functional monomers may lead to the self-association of some functional monomers rather than combination with 2,4-D. In addition, the binding energy of 2,4-D–2,4-D was −62.56 kJ/mol, which was higher than those of 2,4-D–4-VP (−58.54 kJ/mol) and 2,4-D–DMAEMA (−39.31 kJ/mol). Consequently, using 4-VP or DMAEMA as functional monomers may lead to the self-association of 2,4-D instead of combination with 4-VP or DMAEMA. The binding energy of 2,4-D–AM was −61.82 kJ/mol, which was higher than that of AM–AM (−47.87 kJ/mol). Thus, AM may be more easily combined with 2,4-D than other functional monomers.

**Figure 4:**
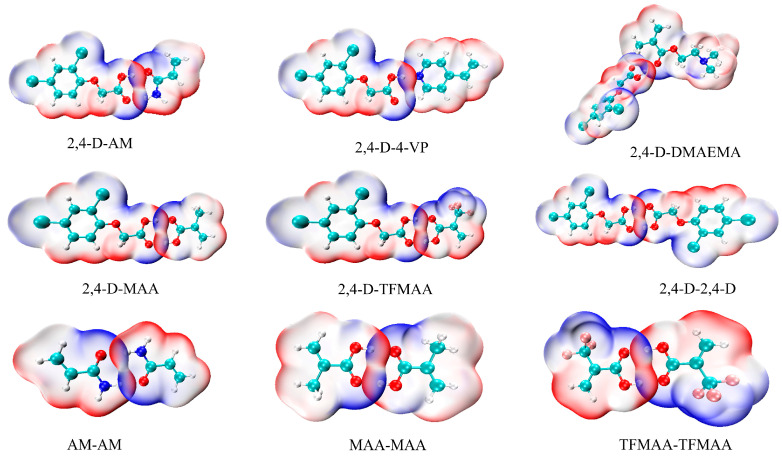
MESP-mapped molecular vdW surfaces of the complexes.

To understand the recognition method between functional monomers and 2,4-D in chloroform, the molecular electrostatic potential (MESP) mapped on molecular van der Waal (vdW) surfaces of 2,4-D and the active monomers was calculated (Figure 4). The variation in the mapped MESP is illustrated using the following coding scheme: red represents the most positive potential regions, blue indicates the most negative potential regions and white area signifies potential halfway between the two extremes of blue and red. Van der Waals surface penetration was visible in the region where hydrogen bonds were formed, and red and blue regions penetrated each other (Figure 4). This indicated the complementary characteristics of electrostatic potential and the essence of the electrostatic attraction interaction of the H-H bond.

### 3.2. MD Simulations for Characterising the Types and Strengths of All Pre-Polymerisation Interactions

#### 3.2.1. Validity of the Force Field and Thermostat

To confirm the validity of the force field, density was considered as a key property. The comparison of experimental and simulated densities at 298.15 K is shown in Table 4. At this temperature, the computed densities were consistent with the experimental values and the error was ~5%, revealing that the constructed molecular force field could reproduce the experimental density. The force field was verified through property computations. In addition, temperature fluctuations within 5 ns of production were recorded (Figure S4).

#### 3.2.2. Imprinting Systems Utilising DMSO as the Solvent

**Figure 5:**
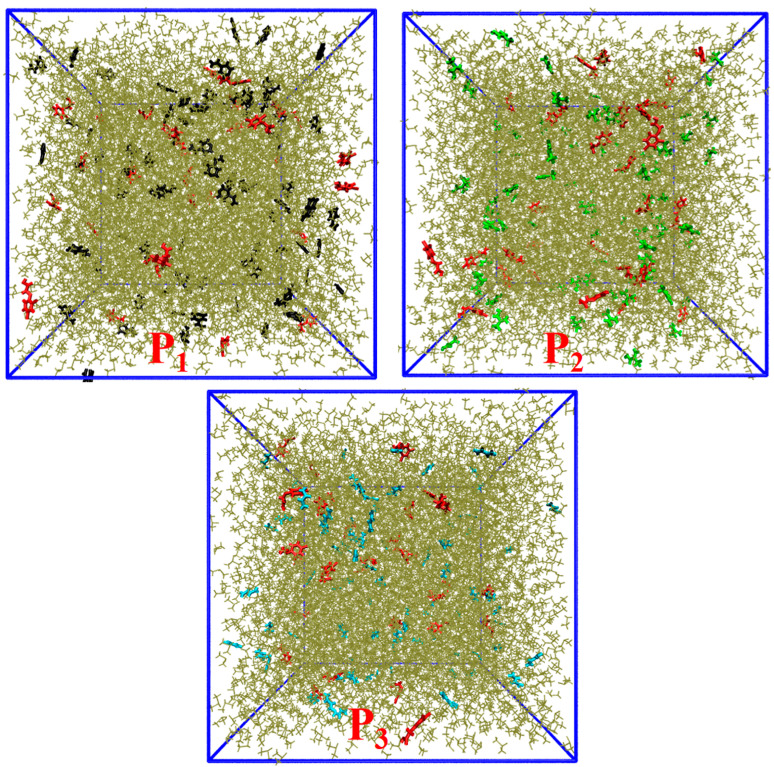
Configurations of the last frames of imprinting systems P1–P3 using DMSO as the solvent. (2,4-D molecules (red); 4-VP molecules (black); TFMAA molecules (green); AM molecules (cyan); DMSO molecules (tan)).

DMSO, a universal solvent, is often selected as an imprinting solvent. The configurations of the last frames in all imprinted systems (P1–P3) with DMSO as a solvent are shown in Figure 5. Numerous DMSO molecules were wrapped with 2,4-D and active monomers. The DMSO molecules in Figure 5 were temporarily masked to enable more obvious observations of the interaction between 2,4-D and the active monomer (Figure S5). The functional monomer and 2,4-D’s hydrogen bond interactions were almost completely disrupted by the addition of DMSO, a protic polar porogen (Figure S5).

To evaluate the interactions between the functional monomer and 2,4-D for each imprinting system using DMSO as a solvent, the total non-bonding interaction was divided into van der Waals (EvdW) and electrostatic (Eelec) (Table 5). In each imprinting system with DMSO, the binding energy between 2,4-D and various functional monomers was small.

**Figure 6:**
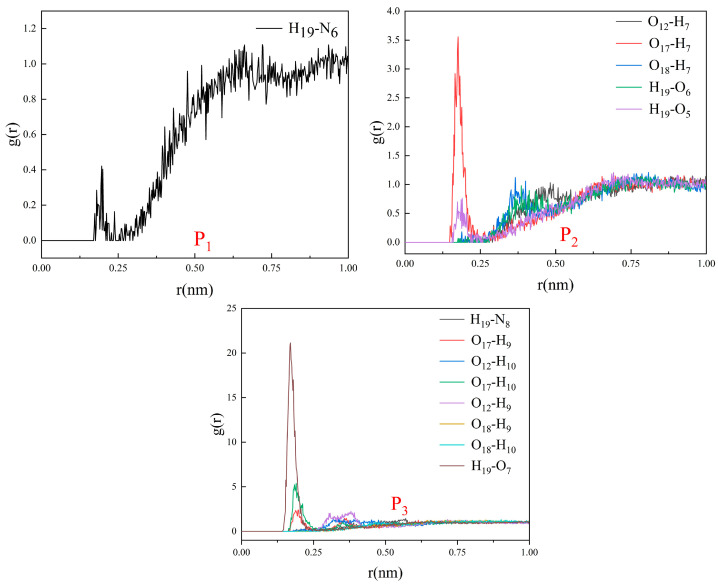
RDFs displaying probabilities of obtaining the atomic densities of the functional monomers at various separation distances from the 2,4-D functional groups in the imprinting systems P1–P3.

RDFs were used to determine the average radial distribution of functional monomers around 2,4-D based on the trajectories. The RDFs indicated that the various functional monomers had a low distribution density in the vicinity (<3.0 Å) of 2,4-D and were almost in the average distribution state in the system (Figure 6). Furthermore, no hydrogen bonds were formed between the functional monomers and 2,4-D (Figure S6). The 2,4-D was not well combined with functional monomers, displaying the free dispersion state, because the polar solvent molecule, DMSO, significantly interfered with the electrostatic force, such as hydrogen bonding and electrostatic interaction.

#### 3.2.3. Imprinting Systems Using Chloroform as the Solvent

**Figure 7:**
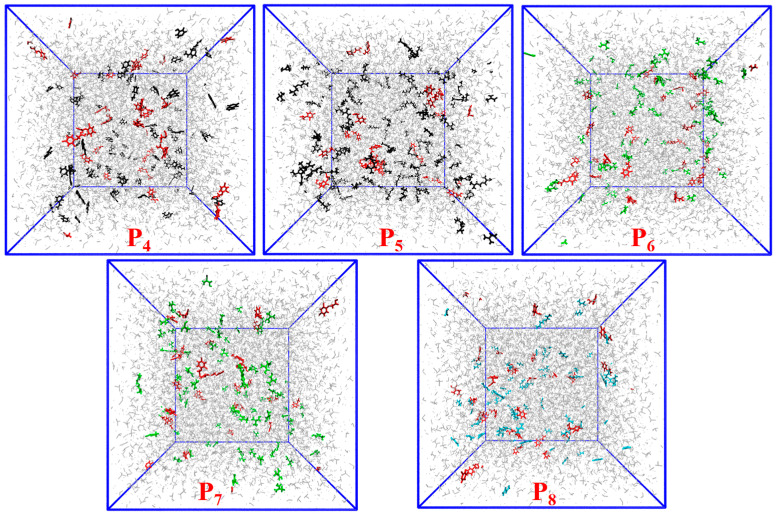
Conformations of the last frames of the imprinting systems P4–P8 with chloroform as the solvent. (DMAEMA molecules (black); 2,4-D molecules (red); 4-VP molecules (black); AM molecules (cyan); TFMAA molecules (green); MAA molecules (green); chloroform (grey)).

Chloroform, a non-polar solvent, is widely employed in the production of non-covalent MIPs. The conformations of the last frames of all imprinted systems (P4–P8) using chloroform as a solvent are shown in Figure 7. To better observe the interactions between 2,4-D and the functional monomers, the chloroform molecules shown in Figure 7 were temporarily masked (Figure S7). The combination of functional monomers and 2,4-D was significantly affected after the solvent was changed from DMSO to CHCl_3_ (Figure S7). Throughout the simulation of 5 ns, AM, MAA and TFMAA exhibited stronger and identical interactions with 2,4-D. DMAEMA was involved in lower levels of binding with 2,4-D and the 4-VP–2,4-D interaction did not exist. The differences in behaviour can be attributed to the relative strengths of the functional groups of the five functional monomers. The primary modes of interaction between 2,4-D and the different functional monomers were further assessed (Figure S8). The binding between the functional monomers and 2,4-D was hydrogen bonding. The 2,4-D, AM, MAA and TFMAA demonstrated a higher degree of dimerisation in the non-polar solvent CHCl_3_ because amide nitrogen and carboxyl groups could form two hydrogen bonds.

The interaction energies between the various functional monomers and 2,4-D in the imprinting systems using chloroform as the solvent are presented in Table 6. To compute the interaction energies, the total non-bonding interaction energies between the functional monomers and 2,4-D were divided into EvdW and Eelec. The non-bonding interaction (−1221.70 kJ/mol) between AM and 2,4-D was significantly stronger than that between 2,4-D and the other functional monomers.

**Figure 8:**
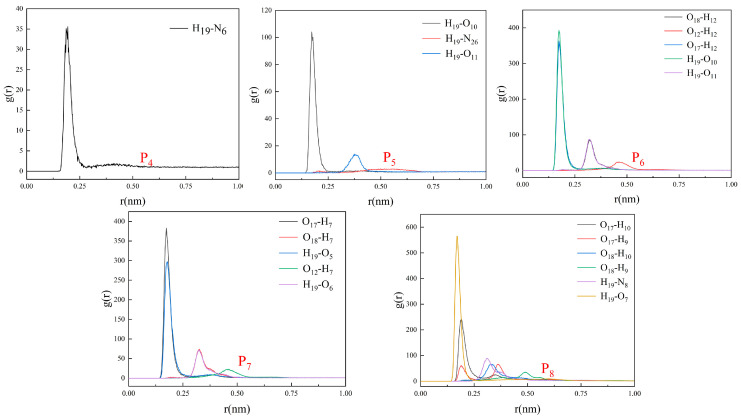
RDFs displaying probabilities of obtaining the atomic densities of the functional monomers for various separation distances from the 2,4-D functional groups in the imprinting systems P4–P8.

**Figure 9:**
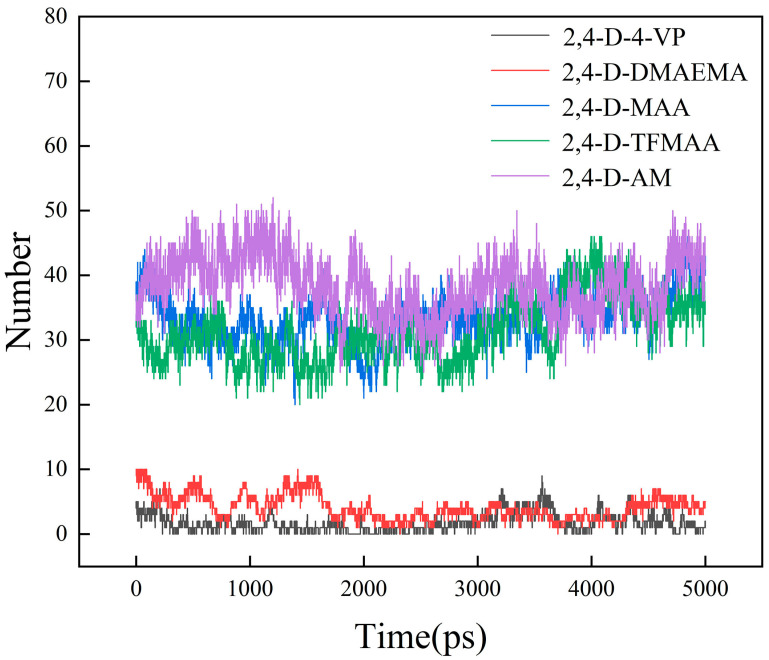
Number of H–H bonds produced between the functional monomers and 2,4-D in the imprinting systems P4–P8 with chloroform as the solvent.

RDFs were used to determine the mean radial distribution of the functional monomers around 2,4-D from the resulting trajectories. RDFs indicated that the probability of AM being close (<3.0 Å) to 2,4-D was higher than the mean probability (Figure 8). The number of H–H bonds formed between the functional monomers and 2,4-D is shown in Figure 9. AM formed a significantly higher number of H–H bonds with 2,4-D (~45) than those formed by the other functional monomers. Meanwhile, very few hydrogen bonds (<5) were formed between 2,4-D and the basic functional monomers.

To examine the spatial distribution of AM, the SDF of AM around 2,4-D was statistically analysed (Figure S9). The density grid representations revealed that the carboxyl group was the primary binding site of 2,4-D in hydrogen bond formation.

#### 3.2.4. Effect of the Ratio of the Template to Functional Monomer

**Figure 10:**
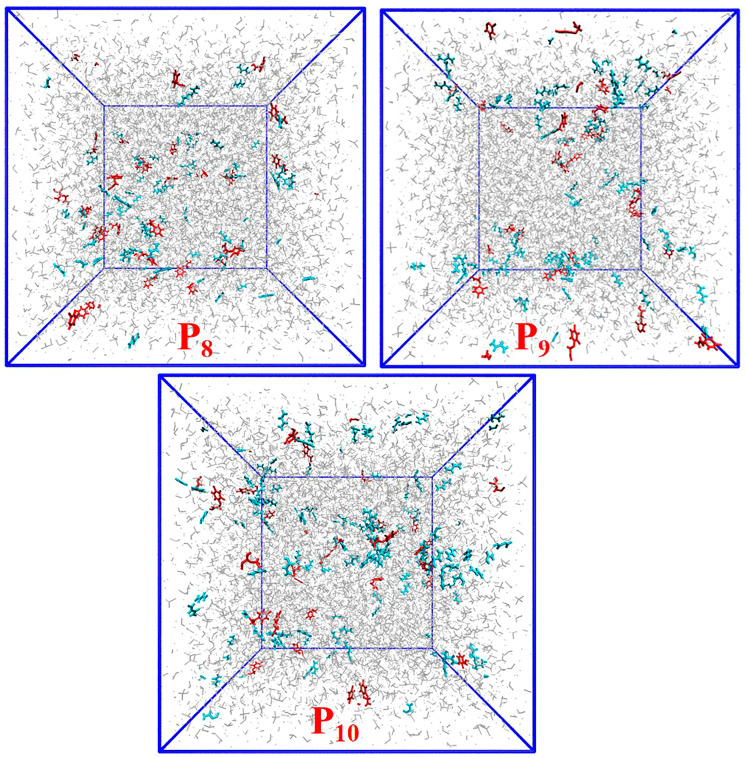
Conformations of the last frame of the imprinting systems P8–P10. (2,4-D molecules (red); AM molecules (cyan); chloroform molecules (grey)).

**Figure 11:**
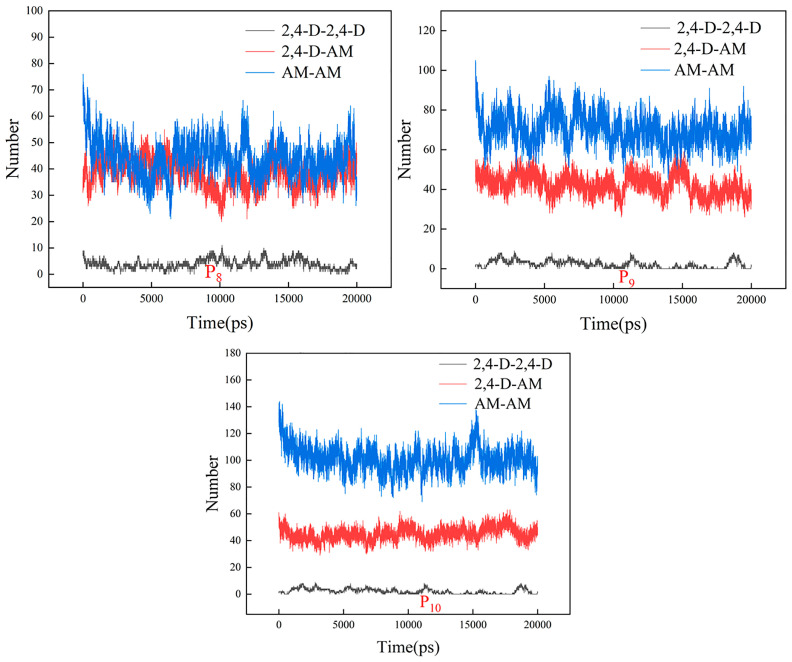
Number of H–H bonds produced between functional monomers and 2,4-D in the imprinting systems P8–P10.

The molar ratio of the template to functional monomer significantly influenced the formation of the template–functional monomer complexes. This study further investigated the effects of the ratio of 2,4-D to AM based on the P8 imprinting system. Three ratios (namely 1:3, 1:4 and 1:5) were selected. The conformations of the last frames of the imprinting systems (P8–P10) are shown in Figure 10. For an enhanced observation of the interactions between 2,4-D and AM, the chloroform molecules in Figure 10 were temporarily masked (Figure S10). As the AM content increased, the amount of free AM in the systems also increased, leading to the formation of non-specific adsorption sites. The number of hydrogen bonds formed between 2,4-D and AM in the imprinting systems (P8–P10) is shown in Figure 11. With the increase in AM content, the number of hydrogen bonds formed between AM and 2,4-D did not increase significantly, but increased among AM and AM (Figure 11).

## 4. Discussion

In this study, a screening method of imprinting systems dependent on a combination of QM computation and MD simulation was used to screen appropriate 2,4-D imprinting systems. The conclusions were drawn from a series of statistical analyses of the molecular trajectories. The polar solvent DMSO interferes with and destroys the weak interaction between 2,4-D and functional monomers to a certain degree, making it an unsuitable imprinting solvent. Conversely, chloroform solvent facilitated the formation of strong bonds between 2,4-D and the functional monomers. AM and various acidic functional monomers formed stronger bonds with 2,4-D than with basic functional monomers (4-VP and DMAEMA). In this study, the theoretical analysis of molecular imprinted systems should account for all types of interactions, including monomer–monomer, template–template and solvent–template, as well as molecular conformational effects to fully explain a system. The proposed method provides a foundation for feasible MIP designs.

## Figures and Tables

**Table 1:** System design and molecular dynamics methodology used.

System	Template	Functional Monomer	Solvent	Pord. (ns)
P_1_	2,4-D (30)	4-VP (90)	DMSO (5597)	5
P_2_	2,4-D (30)	TFMAA (90)	DMSO (5597)	5
P_3_	2,4-D (30)	AM (90)	DMSO (5597)	5
P_4_	2,4-D (30)	4-VP (90)	Chloroform (5597)	5
P_5_	2,4-D (30)	DMAEMA (90)	Chloroform (5597)	5
P_6_	2,4-D (30)	MAA (90)	Chloroform (5597)	5
P_7_	2,4-D (30)	TFMAA (90)	Chloroform (5597)	5
P_8_	2,4-D (30)	AM (90)	Chloroform (5597)	20
P_9_	2,4-D (30)	AM (120)	Chloroform (5597)	20
P_10_	2,4-D (30)	AM (150)	Chloroform (5597)	20

**Table 2:** Interaction energies of 2,4-D–functional monomer complexes in chloroform; 1 kcal/mol = 4.182 kJ/mol.

Complexes	ΔE (kcal mol^−1^)	ΔE (kJ mol^−1^)
2,4-D-MAA	−15.22735846	−63.6808128
2,4-D-4-VP	−13.99844915	−58.5415141
2,4-D-TFMAA	−15.06473297	−63.000713
2,4-D-DMAEMA	−9.400457481	−39.3127132
2,4-D-AM	−14.78296843	−61.8223738
AM-AM	−11.44639108	−47.8688072
2,4-D-2,4-D	−14.95939287	−62.5601807
TFMAA-TFMAA	−15.44972291	−64.6107412
MAA-MAA	−15.31651508	−64.0536657

**Table 3:** Interaction energies of 2,4-D–functional monomer complexes in DMSO; 1 kcal/mol = 4.182 kJ/mol.

Complexes	ΔE (kcal mol^−1^)	ΔE (kJ mol^−1^)
2,4-D-4-VP	−13.28510834	−55.5583229
2,4-D-TFMAA	−13.83121146	−57.8421261
2,4-D-AM	−13.43036435	−56.1657835

**Table 4:** Calculated density of organic small molecules under force fields (unit: 1 g/cm^3^).

Molecule	Simulation Value (GAFF)	Actual Value	Error (GAFF)
EGDMA	1.05966	1.051	0.82%
4-VP	0.974502	0.9800	0.56%
Chloroform	1.50788	1.4840	1.58%
DMAEMA	0.968347	0.933	3.65%
2,4-D	1.524	1.563	2.50%
DMSO	1.10964	1.1	0.87%
MAA	1.020	1.015	0.49%
TFMAA	1.267	1.326	4.45%
AM	1.089	1.122	2.94%

**Table 5:** Interaction energies (kJ/mol) of 2,4-D and different functional monomers in DMSO based on MD simulations. Calculated at 298.15 K and a pressure of 1 bar.

Functional Monomers	Electrostatic Interactions (E_elec_)	vdW Interaction (E_vdW_)	Total Nonbonded Interaction (E_total_)
4-VP	−6.05165	−64.0863	−70.13795
TFMAA	−10.4431	−61.7837	−72.2268
AM	−52.1894	−33.9448	−86.1342

E_total_ = E_elec_ + E_vdW._

**Table 6:** Interaction energies (kJ/mol) of 2,4-D and the different functional monomers in chloroform based on MD simulations. Calculated at 298.15 K and a pressure of 1 bar.

Functional Monomers	Electrostatic Interactions (E_elec_)	vdW Interaction (E_vdW_)	Total Nonbonded Interaction (E_total_)
4-VP	−67.6674	−63.1676	−130.835
DMAEMA	−157.803	−98.5257	−256.3287
TFMAA	−896.046	−25.2375	−921.2835
MAA	−966.629	14.0713	−952.5577
AM	−1208.74	−12.9599	−1221.6999

E_total_ = E_elec_ + E_vdW._

## Data Availability

Data are contained within the article.

## Supplementary Materials

The following supporting information can be downloaded at https://www.mdpi.com/article/10.3390/polym16162257/s1, Figure S1. Change in temperature with simulation time. Figure S2. Geometrical hydrogen bond criterion. Figure S3. Optimised conformations of 2,4-D and five commonly used monomers in MIPs; the ab initio mechanical quantum computations were based on DFT at the B3LYP level with the 6-311+G**basis set. Figure S4. Temperature of the imprinting system P8 within 5 ns production time. Figure S5. Conformations of the last frames of the imprinting systems P1–P3 with DMSO as the solvent. (2,4-D molecules (red); 4-VP molecules (black); TFMAA molecules (green); AM molecules (cyan)). To better observe the binding between 2,4-D and the functional monomers, DMSO molecules were temporarily masked. Figure S6. Number of hydrogen bonds formed between 2,4-D and the functional monomers in the imprinting systems P1–P3 using DMSO as the solvent. Figure S7. Conformations of the last frames of the imprinting systems P4–P8 with chloroform as the solvent. (2,4-D molecules (red); 4-VP molecules (black); DMAEMA molecules (black); TFMAA molecules (green); MAA molecules (green); AM molecules (cyan)). To better observe the binding between 2,4-D and the functional monomers, chloroform molecules were temporarily masked. Figure S8. Main mode of the complexes in the last frame of the imprinting systems P4–P8. Figure S9. Three-dimensional grid-density representations of the imprinting system P8. Densities describe the probability and associated lifetimes for finding AM around 2,4-D. Higher contour values for AM are presented in blue. Figure S10. Conformations of the last frames of the imprinting systems P8–P10. (2,4-D molecules (red); AM molecules (cyan)). To better observe the binding between 2,4-D and AM, chloroform molecules were temporarily masked.
