# Supplementary material for: Rational Design of Non-Covalent Imprinted Polymers Based on the Combination of Molecular Dynamics Simulation and Quantum Mechanics Calculations
Source: Polymers (Basel). 2024 Aug 9;16(16):2257. doi: 10.3390/polym16162257 (PMC11360439; doi:10.3390/polym16162257)
Supplement: Supplementary file 1 [file polymers-16-02257-s001.zip › polymers-3059246-supplementary.pdf]

**Supplementary Materials:**

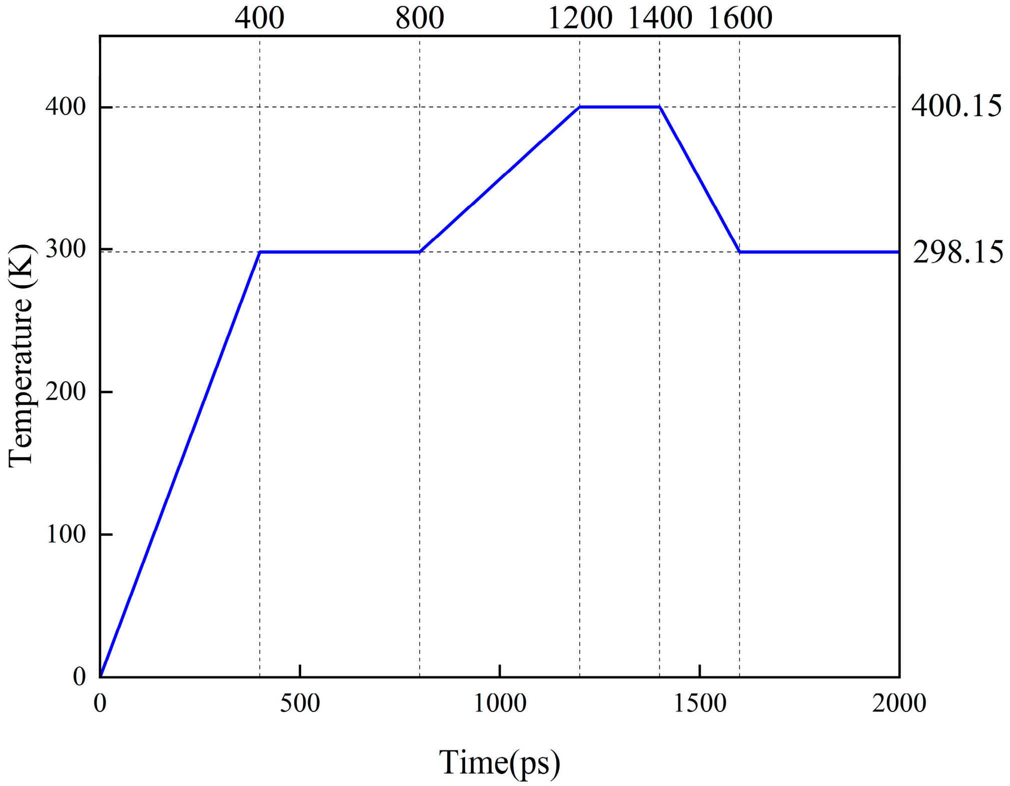


Figure S1. Change in temperature with simulation time.

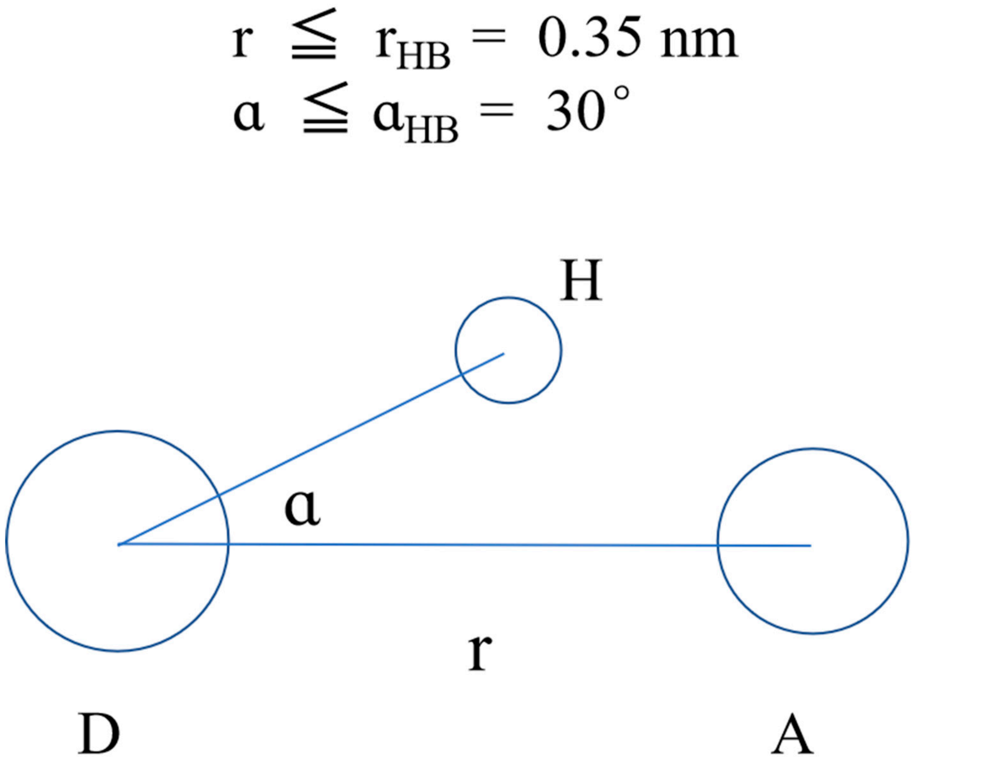


Figure S2. Geometrical hydrogen bond criterion.

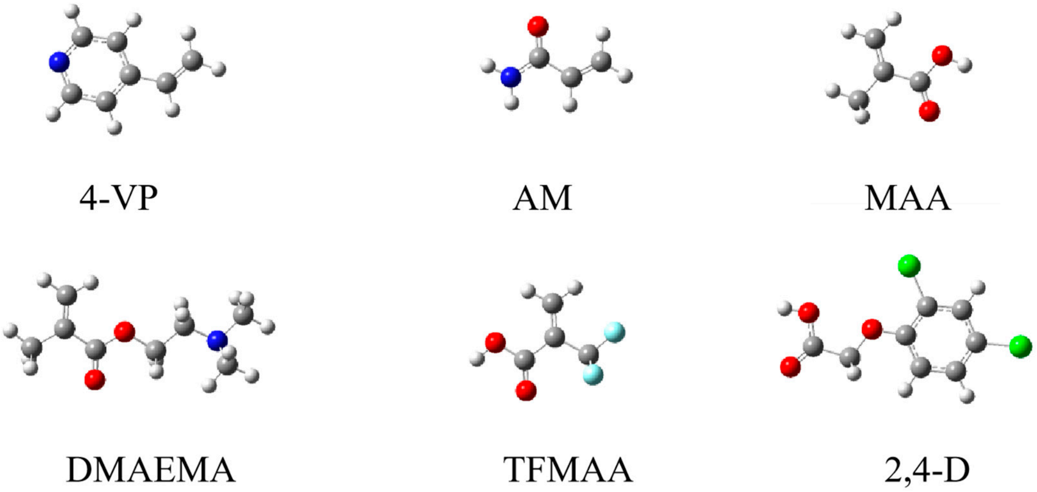


Figure S3. Optimised conformations of 2,4-D and five commonly used monomers in MIPs; the ab initio mechanical quantum computations were based on DFT at the B3LYP level with the 6-311+G**basis set.

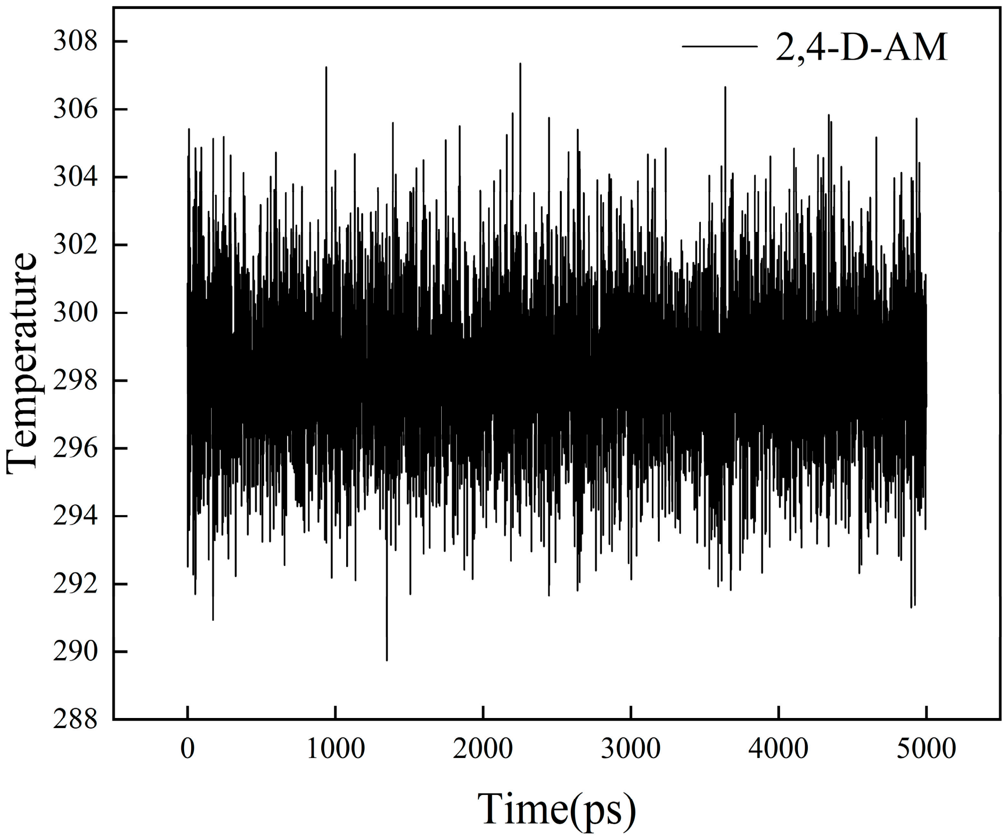


Figure S4. Temperature of the imprinting system P8 within 5-ns production time.

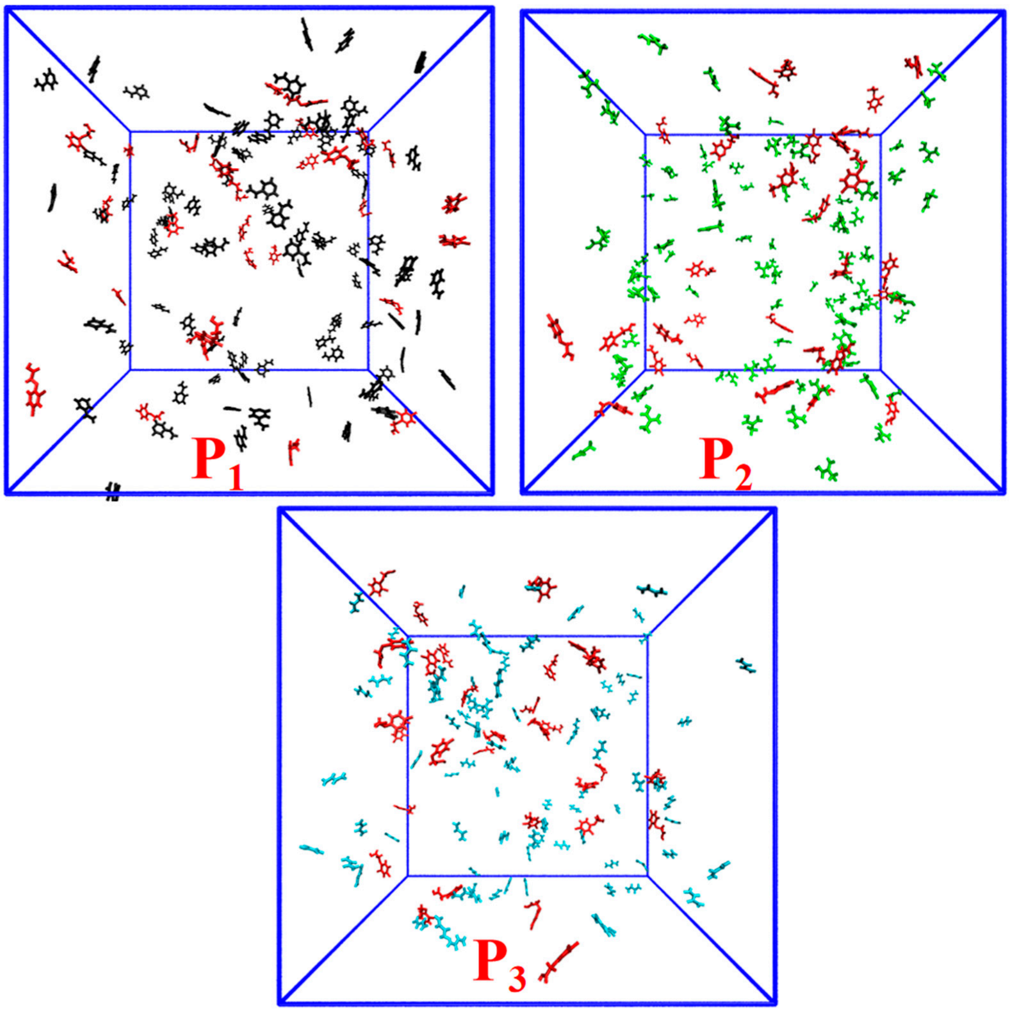


Figure S5. Conformations of the last frames of the imprinting systems P1–P3 with DMSO as the solvent. (2,4-D molecules (red); 4-VP molecules (black); TFMAA molecules (green); AM molecules (cyan)). To better observe the binding between 2,4-D and the functional monomers, DMSO molecules were temporarily masked.

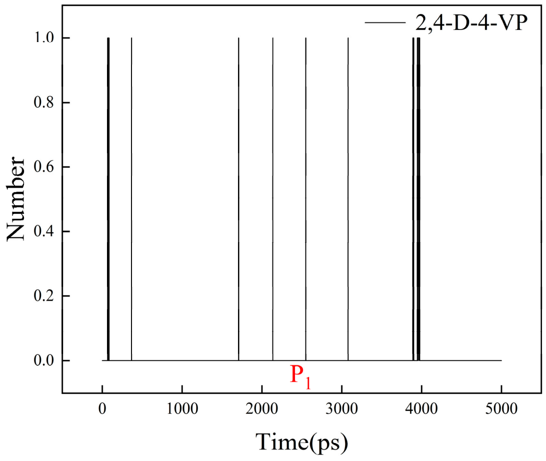


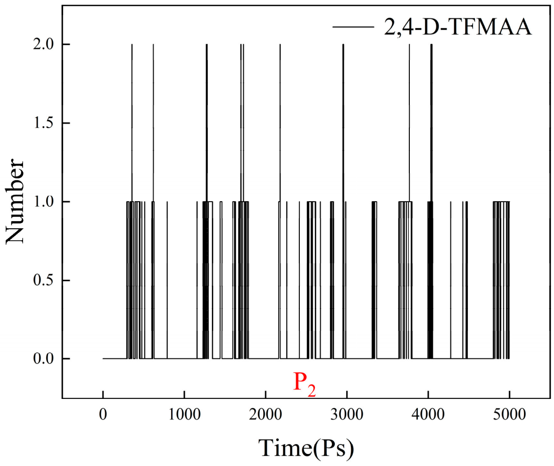

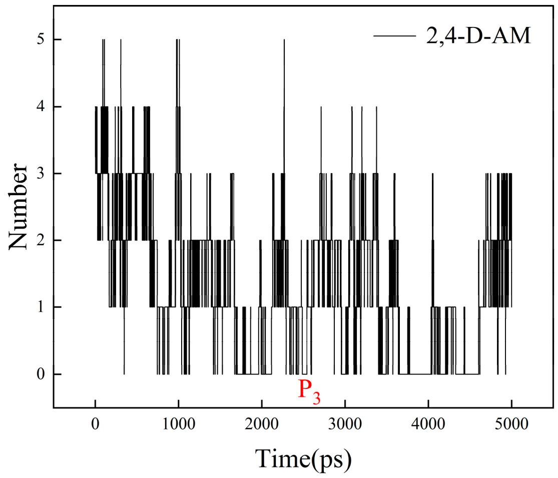


Figure S6. Number of hydrogen bonds formed between 2,4-D and the functional monomers in the imprinting systems P1–P3 using DMSO as the solvent.

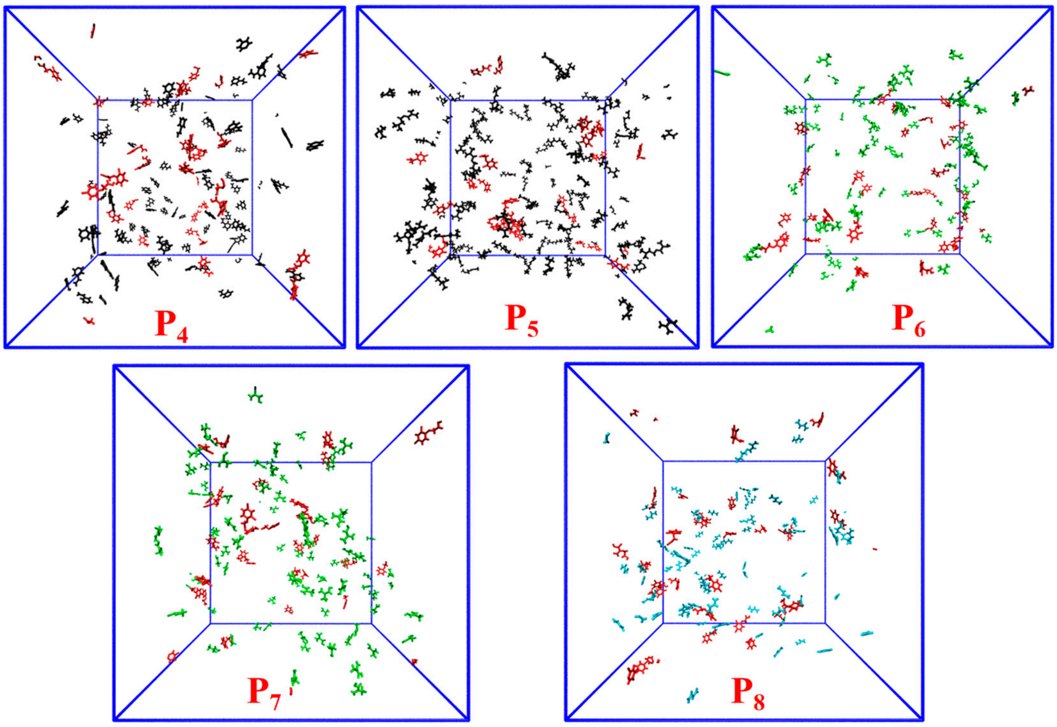


Figure S7. Conformations of the last frames of the imprinting systems P4–P8 with chloroform as the solvent. (2,4-D molecules (red); 4-VP molecules (black); DMAEMA molecules (black); TFMAA molecules (green); MAA molecules (green); AM molecules (cyan)). To better observe the binding between 2,4-D and the functional monomers, chloroform molecules were temporarily masked.

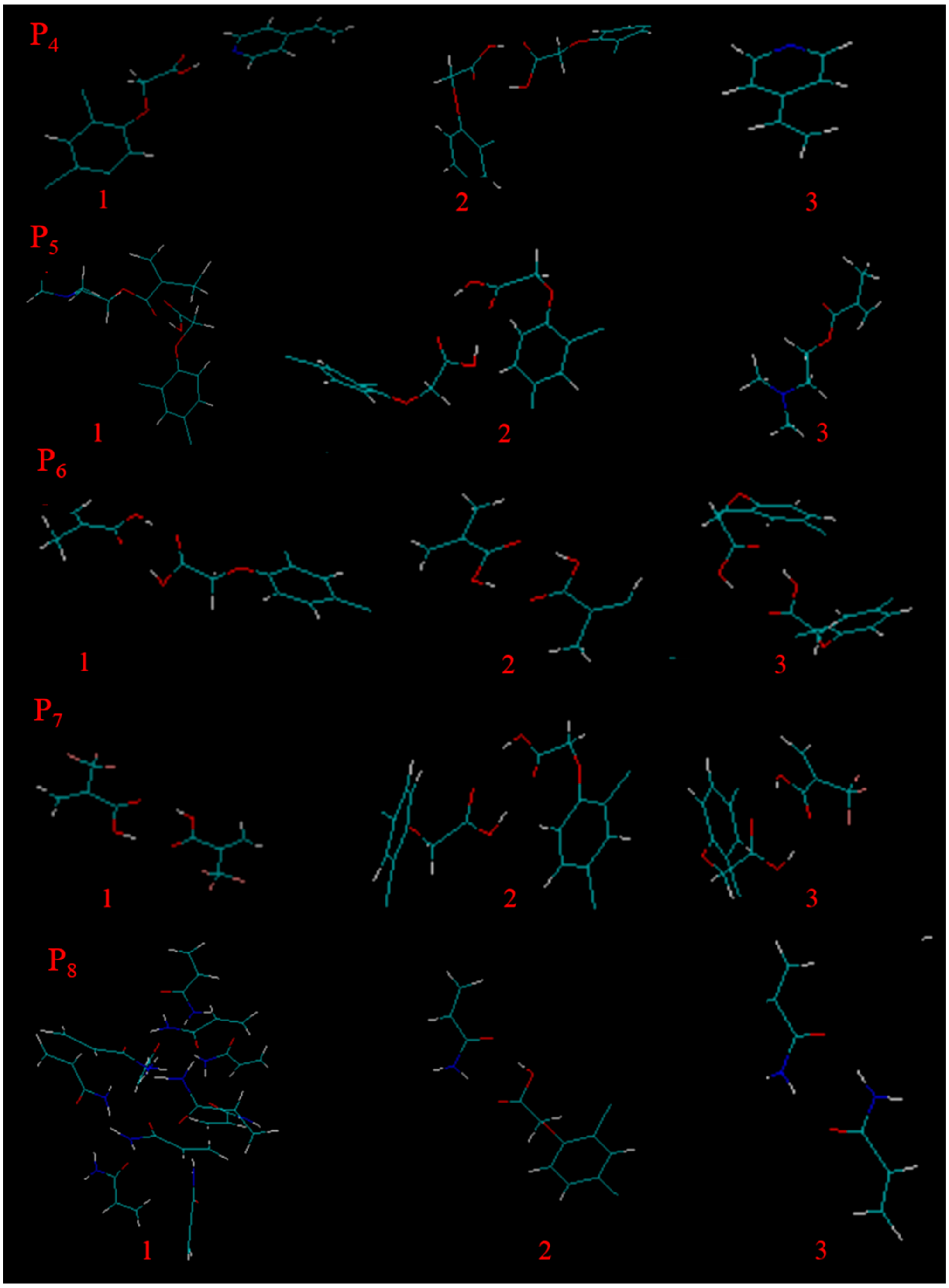


Figure S8. Main mode of the complexes in the last frame of the imprinting systems P4–P8.

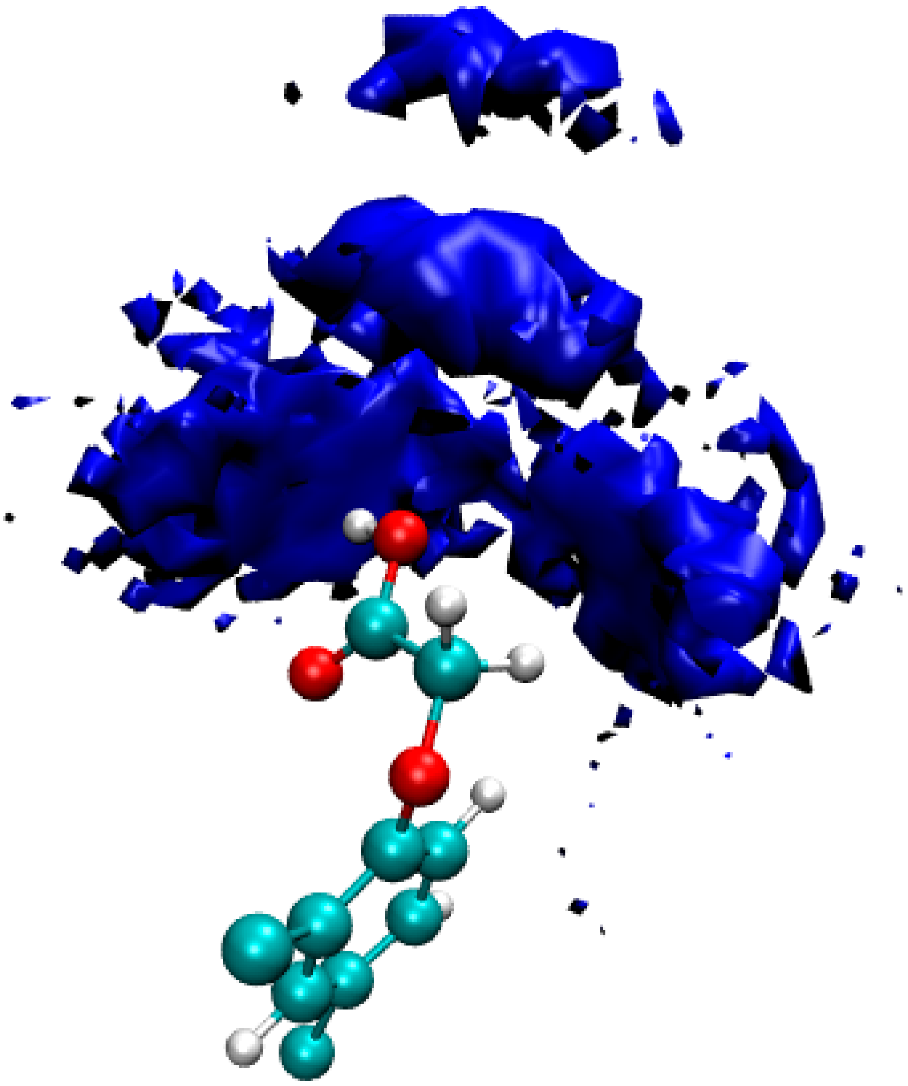

Figure S9. Three-dimensional grid-density representations of the imprinting system P8. Densities describe the probability and associated lifetimes for finding AM around 2,4-D. Higher contour values for AM are presented in blue.

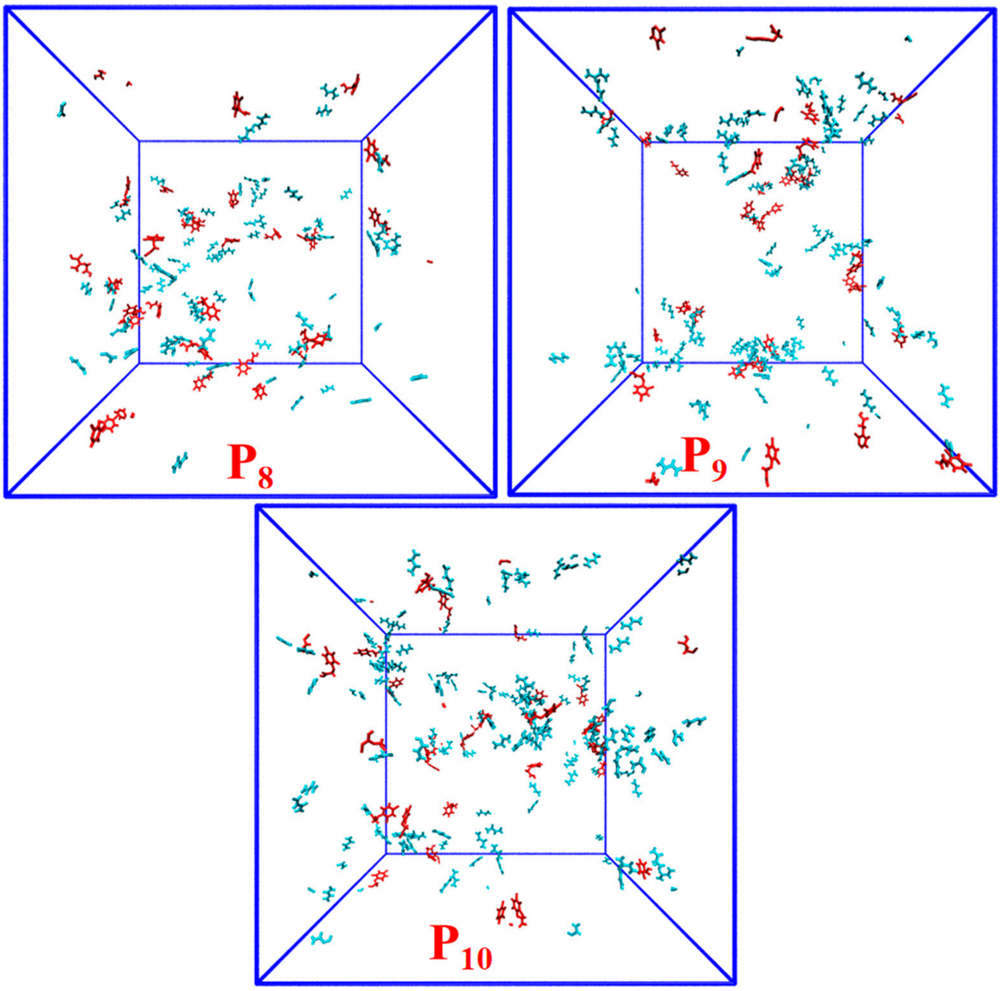


Figure S10. Conformations of the last frames of the imprinting systems P8–P10. (2,4-D molecules (red); AM molecules (cyan)). To better observe the binding between 2,4-D and AM, chloroform molecules were temporarily masked.
